# Microfluidical Microwave Reactor for Synthesis of Gold Nanoparticles

**DOI:** 10.3390/mi8110318

**Published:** 2017-10-26

**Authors:** Jan Macioszczyk, Olga Rac-Rumijowska, Piotr Słobodzian, Helena Teterycz, Karol Malecha

**Affiliations:** 1 Faculty of Microsystem Electronics and Photonics, Wrocław University of Science and Technology, Wybrzeże Wyspiańskiego 27, 50-370 Wrocław, Poland; jan.macioszczyk@pwr.edu.pl (J.M.); olga.rac-rumijowska@pwr.edu.pl (O.R.-R.); helena.teterycz@pwr.edu.pl (H.T.); 2 Faculty of Electronics, Wrocław University of Science and Technology, Wybrzeże Wyspiańskiego 27, 50-370 Wrocław, Poland; piotr.slobodzian@pwr.edu.pl

**Keywords:** microfluidics, microwave chemistry, low temperature co-fired ceramics (LTCC)

## Abstract

Microwave treatment can reduce the time of selected syntheses, for instance of gold nanoparticles (AuNPs), from several hours to a few minutes. We propose a microfluidic structure for enhancing the rate of chemical reactions using microwave energy. This reactor is designed to control microwave energy with much higher accuracy than in standard devices. Thanks to this, the influence of microwave irradiation on the rate of chemical reactions can be investigated. The reactor consists of a transmission line surrounded by ground metallization. In order to deliver microwave energy to the fluid under test efficiently, matching networks are used and optimized by means of numerical methods. The monolithic device is fabricated in the low temperature co-fired ceramics (LTCC) technology. This material exhibits excellent microwave performance and is resistant to many chemical substances as well as high temperatures. Fabrication of the devices is described in detail. Measurements of microwave parameters are performed and differences between simulation and experiment results are discussed. Finally, the usefulness of the proposed device is proved in exemplary synthesis.

## 1. Introduction

Microwaves are a time and space varying electromagnetic field. Their frequencies lay in the range that is typically defined from 300 MHz to 300 GHz. They are commonly used and find their main application in wireless communication. However, microwaves are also used for other purposes because of their interactions with matter. Probably, the most common is for food heating in a microwave oven. This heating phenomenon can also be applied for cancer treatment [1]. Applications of microwave irradiation in chemistry have been widely reported in the literature [2,3]. Microwave treatment can be applied for enhancing synthesis of organic [4] and inorganic materials [5], as well as nanomaterials [6].

Recently, examples of microwave microfluidic devices were presented. They typically serve for sensing or heating samples (biological or chemical). The device ability for characterization of liquid solution is based on the fact that various materials have different electric and magnetic properties. The intrinsic parameters that describe the reaction of a material on the external electromagnetic field are the permittivity and the permeability. Therefore, measurements of these parameters give us information about the properties of the material under test (MUT). The permittivity and permeability can be obtained in the microwave range by observing the input impedance of a transmission line, which is related to the MUT, or power transmitted to the MUT. The first method can be used in a wide range of frequencies, and the second one only in narrow frequency bands, but with higher accuracy. Microfluidic devices for such applications have been fabricated as external devices connected with standard microwave structures [7,8], or integrated systems [9,10].

The second class of microwave microfluidic devices is used for transferring microwave energy to fluids, particularly in order to heat them. The advantage of microwave heating over conventional heating is threefold. Firstly, this way of changing temperature can be beneficial because energy can be selectively delivered to the fluid inside the reactor. Thanks to low dielectric losses of the low temperature co-fired ceramics (LTCC) the energy of microwaves mainly is dissipated in the fluid. As a consequence, only fluid in the reactor is heated, not the entire device. Secondly, microwave heating can be precisely controlled. By measuring a fraction of microwave power that is reflected at the input port of a microwave reactor, and power that is transmitted through the reactor, we can determine power that is absorbed by a sample inside this reactor quite accurately. This is not the case when the conventional heating is considered. In that case, it is very difficult to separate power absorbed by the body of the reactor itself and by the sample inside the reactor. Thirdly, when transmitting microwave energy (a signal) through a reactor we can obtain additional information about a sample placed inside this reactor. A microwave response of such a system contains information that is specific to this sample. It is possible to extract electrical parameters of the sample accurately, and those parameters are correlated (quite strongly, often) with physical and chemical properties of this sample.

The most intuitive way to deliver microwave energy is to treat the sample as a load. This method has been successfully implemented by a few research groups and used, for example, for DNA amplification [11,12]. Despite its advantages, such as simple hardware implementation and good heating rates, the method has two main drawbacks. Firstly, it is difficult to specify the power budget accurately, since the microfluidic device operates as a one-port microwave network. Moreover, it is very hard to establish the ratio between power dissipated in the sample and in the whole device. Secondly, such systems work efficiently for selected and relatively narrow frequency bands. Thus, for changing the operating frequency of the device, a new design is required.

The other approach is to combine the microfluidic channel with a microwave transmission line, which can assume the form of a coupled line [13], a coplanar waveguide [14], or a microstrip line [15]. In all those cases the usable range of frequency is much wider than in the previous case. What is more, a transmission line has two ports (the input and output port); thus, we can measure not only the reflected but also the transferred power. This enhances the accuracy of the determination of power dissipation. However, heating rates are generally lower in comparison to one port devices.

In this article, we propose a novel microwave microfluidic reactor that is based on LTCC. This technology was primarily developed to increase the capability of the thick-film technology, especially for applications in mainboards and high-frequency circuits [16]. The LTCC materials are well characterized in a microwave range [17,18,19,20]. Research made on this technology allowed us to use a variety of materials and to fabricate sensors [21], microsystems [22], and microfluidic devices [23]. During our experiments, all those possibilities were taken into account. As a result, a novel LTCC-based device (a microwave reactor) with integrated microfluidic, and microwave components was proposed. The design, fabrication process, and properties of such a device are described. The performance of the proposed microwave reactor was assessed during the synthesis of gold nanoparticles (AuNPs).

Gold nanoparticles, due to their unique properties, can be used in many fields, such as electronics, catalysis [24], or medicine [25]. For this reason, there is still an increasing interest in methods for obtaining nanoparticles. The simplest and most common way to obtain gold nanoparticles is chemical synthesis. In 1857, Faraday described the synthesis of gold nanoparticles and attributed them to the color red [26]. AuNPs are usually obtained by reducing gold ions from the precursor, which is chloroauric acid. Colloidal solutions of nanoparticles are thermodynamically unstable and agglomerate. As a result of this process they lose their unique properties. For this reason, it is necessary to use stabilizers, which are usually polymers such as polyethyleneimine (PEI) [27], polyvinylpyrrolidone (PVP) [28], or polyethylene glycol (PEG) [29]. Gold ions can be reduced by using citric acid, ascorbic acid, or sodium borohydride [30]. It is also possible to obtain nanoparticles in the presence of a stabilizing polymer without the use of reducing agents [31]. However, in such cases, the synthesis takes several days or weeks [27,32]. The way to reduce the reaction time without a reducer can be to conduct the process in the microwave field. The gold ion reduction reaction, carried out in the presence of microwaves, is much faster [33]. In some cases, microwaves do not replace the reducing agent in all processes, and in some cases microwaves only support them [34]. Augustine and others obtained gold nanoparticles as a result of the reduction with sodium citrate in the presence of microwaves. However, the nanoparticles agglomerated when no stabilizing agent was used [35]. Gutiérrez-Wing and others received gold nanoparticles in a two-phase system in the presence of microwaves that spontaneously aggregate into self-supported superstructures [36]. In most cases, when gold nanoparticle synthesis was provided without stabilizing agents, AuNPs agglomerated rapidly.

This paper presents the synthesis of PEI stabilized gold nanoparticles. Gold ions were reduced only under the influence of the microwave field. The process was carried out in an LTCC reactor.

## 2. Materials and Methods

### 2.1. Benchmark Reaction

In order to examine the utility of the proposed reactor we had to find the chemical reaction that is significantly affected by the microwave irradiation. The progress of the selected reaction was examined visually and by ultraviolet–visible (UV-vis) spectroscopy. For this reason, we chose the gold nanoparticles synthesis, which is described in [37]. 

Firstly, 100 mL of deionized water and 0.4 mL of high molecular polyethyleneimine (PEI) as a stabilizer was mixed. Then, after a few minutes of mixing, 0.312 mL of tetrachloroauric acid (HAuCl_4_) was added. Next, a portion of this mixture was placed in a microwave oven for a certain time. The mixture was in a glass, open vessel to keep atmospheric pressure during the reaction. To decrease the influence of rising temperature, the power was set to the lowest possible value. In fact, during our experiment, all samples under test never reached boiling point.

**Figure 1:**
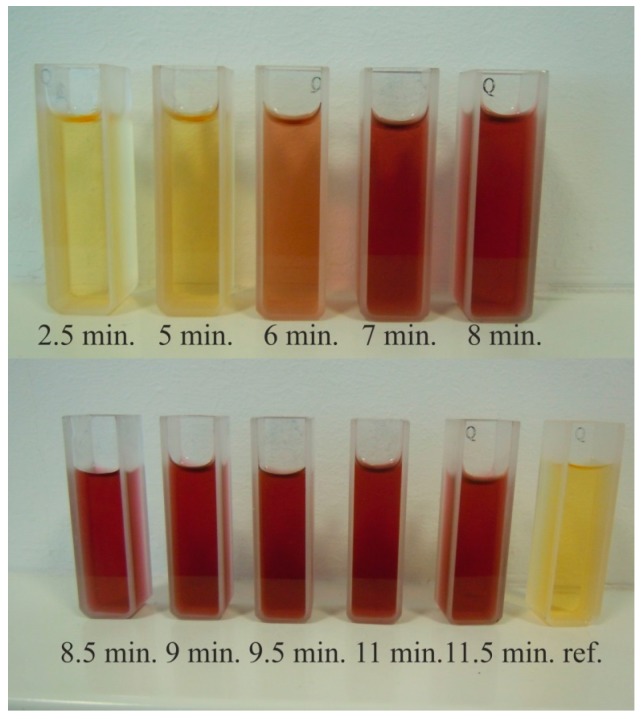
Samples after a certain time of microwave treatment.

**Figure 2:**
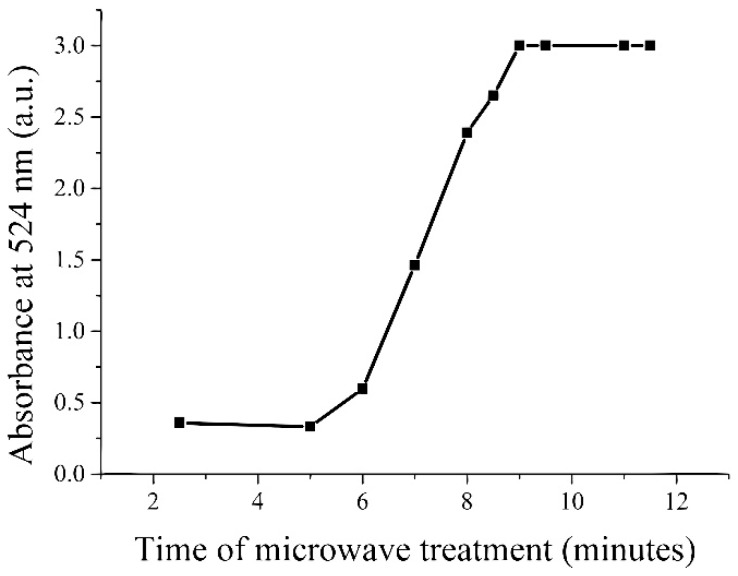
Intensity of samples absorbance at 524 nm.

The samples after microwave treatment are presented in Figure 1. To obtain quantitative information about synthesis efficiency we did absorbance measurements using a spectrophotometer (Mecasys Optizen Alpha, Mecasys Co., Ltd., Daejeon, Korea). The number of synthesized particles was indicated by the intensity of the absorbance peak at a wavelength of 524 nm (see Figure 2). One may observe that reactions significantly accelerated after 5 min of microwave treatment. Also, the synthesis was completed after approximately 9 min. Reference sample needed more than 8 h of mixing at room temperature to have similar absorbance spectra. 

### 2.2. Design of the Microreactor

**Figure 3:**
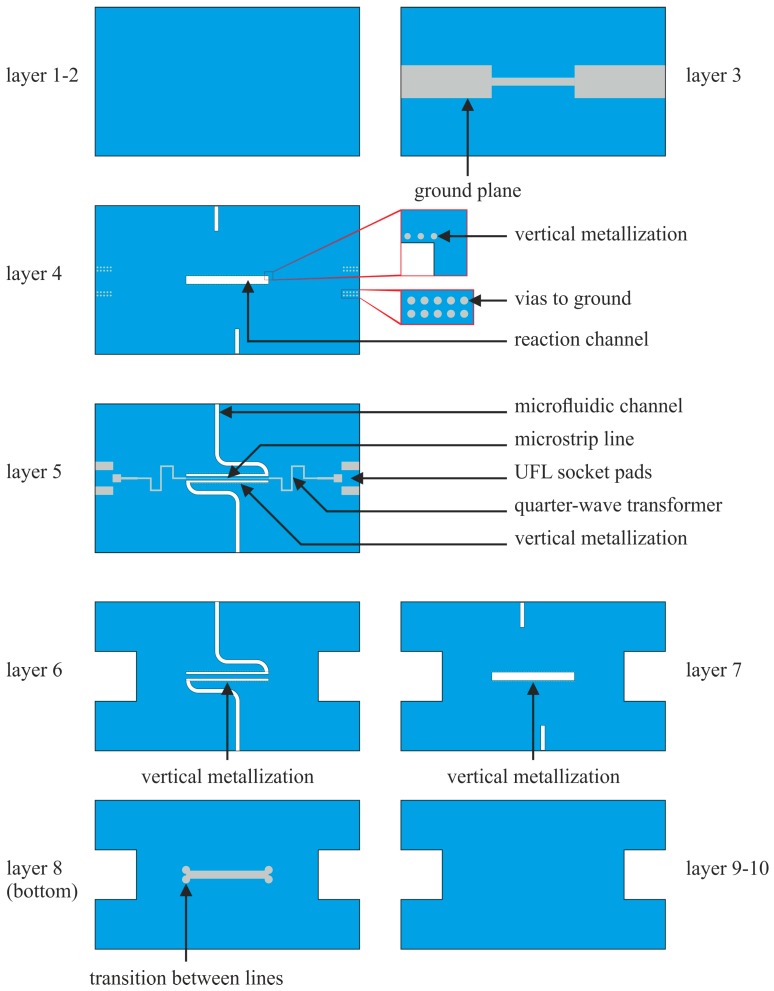
Design of the low temperature co-fired ceramics (LTCC) layers to construct the microfluidical microwave reactor.

The proposed monolithic device for microwave treatment was fabricated in the LTCC technology (DuPont 951 system, DuPont, Wilmington, DE, USA). A schematic view of the layers are shown in Figure 3. Most important is the middle part (layer 5). It consists of pads for the U.FL (ultra-small surface mount coaxial connector) connectors mounting, two matching networks, and a stripline-like transmission line. The transmission line is embedded in a microfluidic channel (layers 4–7). The signal line is placed inside the 200 µm thick and 400 µm wide ceramic strip. The strip was suspended in the middle of the reactor. The transmission line is surrounded by the fluid during the reactor operation. The ground planes are placed below (layer 3) and above (layer 8) the ceramic strip at a distant of 213 µm from it (one DuPont 951PX tape, DuPont, Wilmington, DE, USA). They are connected by vertical metallization (layers 4–7). The metallization also covers sidewalls of the channel, through which microwave energy is transmitted, so the rest of the LTCC module is screened electromagnetically. Also, to improve mechanical properties of the structure, three layers of 951PX tape were placed above (layers 1–2) and below (layers 9–10) the ground planes. To our best knowledge, this kind of structure is presented for the first time. Its advantage is that one can precisely control the microwave energy delivered to the reactor, because, independently of the permittivity of the fluid under treatment, the electric field always crosses the microfluidic channel in a way that allows microwave energy to be transmitted through it. Our structure was designed to operate at industrial scientific medical (ISM) 2.4 GHz band. We used the high frequency electromagnetic field simulation (HFSS) software (version: 14, ANSYS Inc., Canonsburg, PA, USA) for the CAD design.

In order to deliver microwave power efficiently, two problems had to be solved. The first one was a transition between different types of the transmission line. The microstrip line and stripline support different modes of the electromagnetic (EM) field associated with microwave energy propagation. In order to avoid power reflections from interfaces between two different lines, a suitable transition between them had to be created. Based on the solution presented in [38], a special shape of metallization on the bottom layer of the reactor was designed (layer 8). The second problem was associated with the impedance mismatch, which occurred when the reactor was filled with a fluid sample. For example, pure water has approximately ten times higher dielectric constant than the LTCC ceramics. In order to obtain 50 Ω impedance of the stripline, its width had to be lower than 10 µm. Such a width exceeds limitations of the thick-film technology. Therefore, two quarter-wave transformers in form of folded line have been proposed. The transformers length and width were optimized to get good matching in a possibly wide frequency range when transmission line had a width of 120 µm.

**Figure 4:**
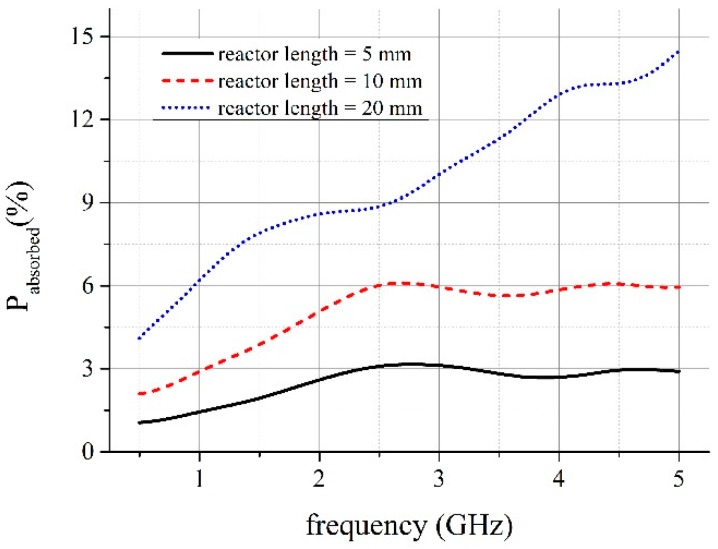
Expected power absorption in the reactor.

The final dimensions of the reactor were 1 mm in width and 0.63 mm in thickness. Based on the numerical simulations, we assessed the expected power dissipation in reactors having different length, i.e., 5, 10, and 20 mm. Different lengths, as well as good impedance matching, were necessary to fulfill requirements of a method used for the determination of power absorbed by the sample under test. A theory related to this method will be explained in Section 3.2. The expected (simulated) power absorption in function of frequency is presented in Figure 4. The absorption is calculated on the assumption that water properties are constant during operation of the reactor. It is not exactly true in the real case because power absorbed in the reactor increased the temperature of water. This would cause a change of water permittivity and, as a result, impedance mismatch. This effect would also depend on flow rate. However, numerical simulation of the coupled fields is a complex issue; therefore, at this stage, we had to proceed our investigations with making such simplifications.

## 3. Results

### 3.1. Microreactor Fabrication

Our devices were fabricated in DuPont 951 ceramic substrates with dielectric constant 7.8 and loss tangent 0.006. In the first step, after preconditioning, tapes were cut into smaller pieces using LPKF ProtoLaser U (Nd:YAG 355 nm, LPKF Laser & Electronics AG, Garbsen, Germany). In the same process vias for electrical connection and opening for stacking and lamination were done. Such processed ceramic tapes were ready for the screen-printing process. To create precise patterns 400 M screen and ESL 903a paste (ESL ElectroScience, King of Prussia, PA, USA) were used. DuPont silver paste for ground planes (LL602, DuPont, Wilmington, DE, USA) was deposited by 325 M screen in order to make the bottom and top metallizations. Vertical metallizations of the reactor were fabricated as vias with 100 µm diameter and 200 µm distances between their centers in layers 4–7. It is typical solution used in microstrip circuits to “imitate” continuous vertical layers. All vias were filled with ESL 902 paste.

In the next step, two 114 µm-thick LTCC tapes (layers 5 and 6 in Figure 3), i.e., one with the middle layer pattern and the second for burying the transmission line, were laminated in the standard isostatic process (20 MPa at 70 °C during 10 min). The LTCC tapes for microfluidic channels and reactor were cut using a laser. It was a crucial step because an improper set of laser parameters could cause local overheating of ceramics. As a result, because of different properties of vias and green ceramics, mechanical stress could damage reactor. 

**Figure 5:**
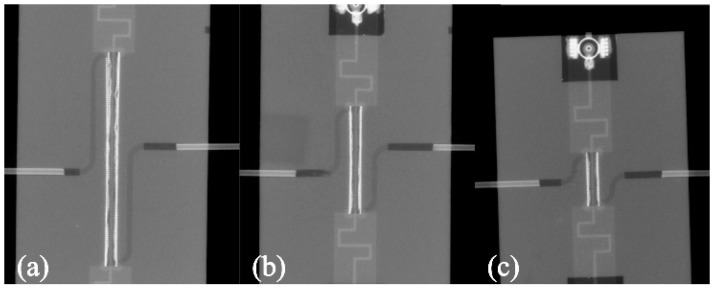
X-ray images of (**a**) 20 mm, (**b**) 10 mm, and (**c**) 5 mm long microreactor.

Then they were stacked with two other tapes (namely layers 4 and 7 in Figure 3) of the reactor (both 254 µm in green state) and laminated under reduced pressure (5 MPa) to avoid sagging of channels and reactor. The places for microfluidic ports were cut in these layers. In the next step, the layers were connected with top (layers 8–10; Figure 3) and bottom (layers 1–3; Figure 3) parts with ground metallizations. Lamination was conducted under pressure of 5 MPa for 15 min. Therefore, we did not have to use any fugitive materials to fabricate the devices. Finally, devices were singulated and fired (T_max_ = 875 °C). Then, the microfluidic ports were glued and UFL sockets were soldered. Three devices with 5, 10, and 20 mm length of the reactor were fabricated. Their volumes were 2.75 µL, 5.5 µL, and 11 µL, respectively. X-ray images of them are presented in the Figure 5.

### 3.2. Electrical Measurements

**Figure 6:**
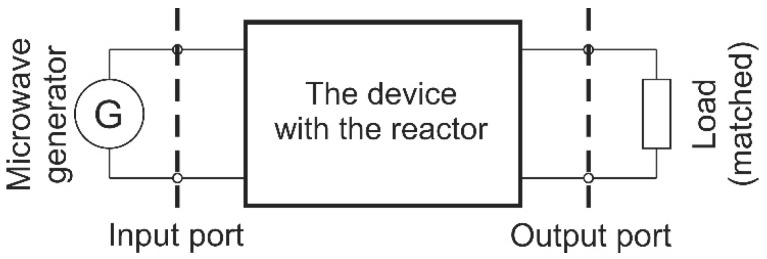
The proposed model of the microwave-powered microreactor.

**Figure 7:**
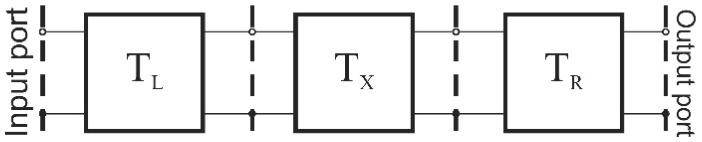
A three-stage cascade network model of the device.

The microwave measurements were provided using vector network analyzer Agilent N5230A (Agilent Technologies, Santa Clara, CA, USA). The reflection and transmission coefficients were measured for an empty reactor and the reactor filled with deionized water. Water was introduced via stainless steel nozzles using a syringe. Measurements were provided up to 5 GHz with 25 MHz resolution. Then, the obtained S-parameters were converted into parameters of ABCD matrix. The power extraction method is based on the ABCD-parameters of a cascade, which describes consecutive parts of the microfluidic-microwave system, which is shown in Figure 6. The cascade consists of three two-port networks, illustrated in Figure 7, where T_L_ and T_R_ stand for chain matrices of the input and output microwave networks, which are used to deliver and receive the microwave power, and T_X_ stands for the chain matrix of the microreactor.

In order to analyze the system, we assume that the radiation loss in this system is negligible, and the EM coupling between the input and output of the system is so small that it can be neglected too. Our aim is to determine T_X_. This matrix allows us to determine the amount of dissipated power; also, it contains information about electrical parameters of a substance inside the microreactor. Unfortunately, the only information that is available about the system is the ABCD matrix of the whole cascade, i.e., T_w_ = T_L_ × T_X_ × T_R_. In order to decrease the number of unknowns, we can make use of measurements of two different cascades, which differ in terms of the length of the microreactor part (T_X_). 

**Figure 8:**
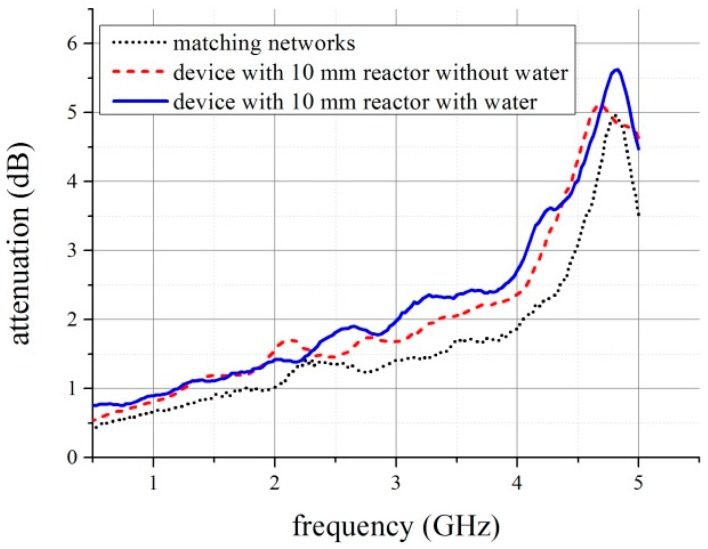
Measured attenuation of the device with and without water.

**Figure 9:**
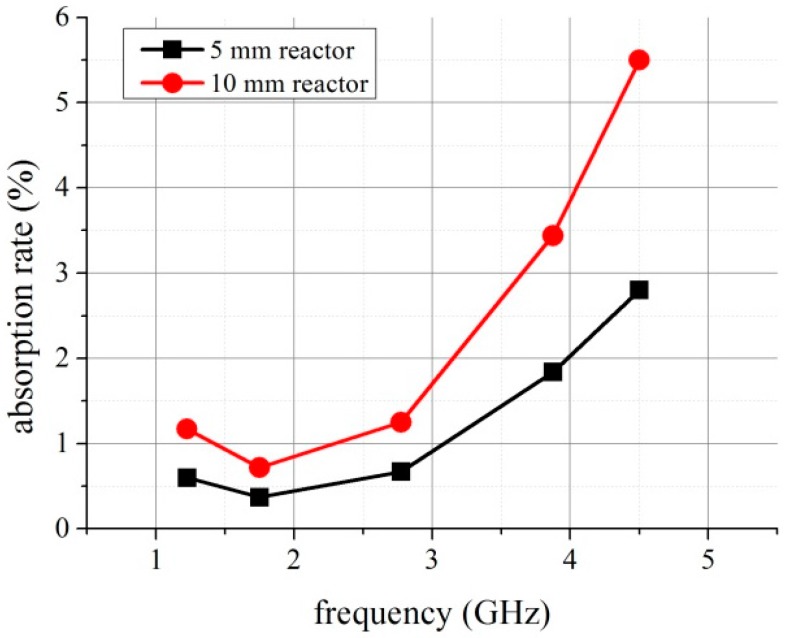
The power absorption rate determined for the reactor part filled with water.

Using the presented theory we have calculated losses in devices and its consecutive parts—both the matching circuits and the reactor. The results obtained for the device with 10 mm long reactor are presented in Figure 8. One may observe that for some frequency ranges, for instance, lower than 2.3 GHz, the reactor absorbed more power when it was empty. This strange result appeared because of unexpected power reflections. Unfortunately, the required impedance-matching condition was not satisfied to allow the dissipated power to be calculated accurately in a broad frequency range. That was the case especially for 20 mm long reactor. Approximated values of power dissipation for shorter reactors are presented in Figure 9. The visible differences will be explained in Section 4.

### 3.3. Nanomaterial Synthesis in the Reactor

**Figure 10:**
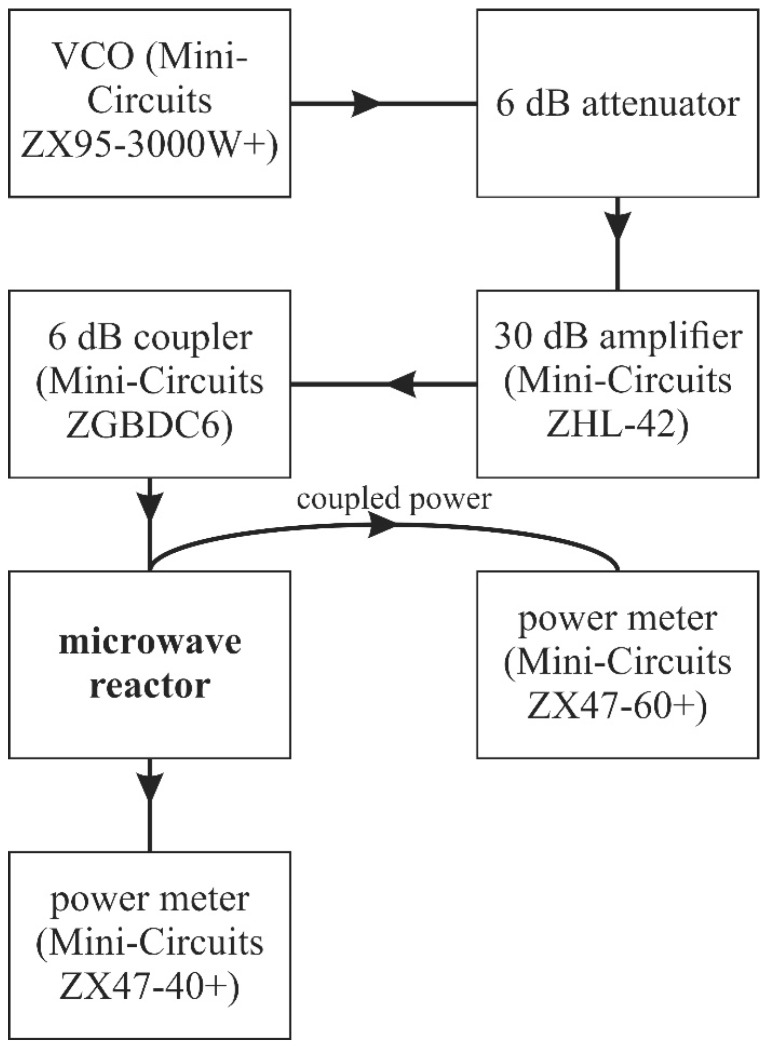
Scheme of setup for sample treatment in microfluidic reactor.

Despite differences between numerical simulations, we have checked if our device can be used for supporting nanomaterial synthesis. Scheme of set-up is presented in Figure 10. We have used a voltage controlled oscillator (VCO), that can be tuned from 2 GHz to 3 GHz. It was connected to the power amplifier through a 6 dB attenuator, not to exceed the maximum input power. Next, was a bi-directional 6 dB coupler, it allowed us to measure power reflected from microwave reactor input. Also, the output of the 10 mm reactor device with was recorded. All of these components have been fabricated by Mini-Circuits (Brooklyn, NY, USA) and their specifications can be found on their webpage [39].

Before the experiment, we prepared the test solution as it was described earlier. In the next step, we start to pump fluid using a peristaltic pump (REGOLO ICC, ISMATEC, Wertheim, Germany). During this measurement flow rate was constant and set to 200 µL/min. Firstly, we checked the amount of power dissipated in the device. In the VCO range of tuning, this power was rather constant—fluctuations of readings in time were more significant than ones caused by the sweeping frequency. If we assume, that, with the exception of the microwave reactor, all parts of the system are perfectly matched and lossless, which seems to be a quite good approximation, then power delivered to the device was equal to 475 mW.

In the next step, we set the frequency to 2.75 GHz. For this value, we obtained a quite accurate value of power dissipated in the reactor equal to 1.25%. According to this, in every second in the reactor 5.94 mJ was absorbed. It is then possible to change the amount of microwave energy by changing the flow rate of the sample, and thus the time when the fluid is inside the reactor.

Then, we tested how the amount of energy influences the synthesis. We set the flow rate to 800 µL/min, 400 µL/min, and 200 µL/min. It corresponds to times in the reactor of 0.41 s, 0.83 s, and 1.65 s, which gives 2.45 mJ, 4.9 mJ, and 9.8 mJ, respectively. If we now assume that the specific heat and density of the sample are the same as water, then the temperature should rise by 0.11 K, 0.21 K, and 0.43 K, respectively. This simplification can be justified because water was more than 99% of the sample volume.

**Figure 11:**
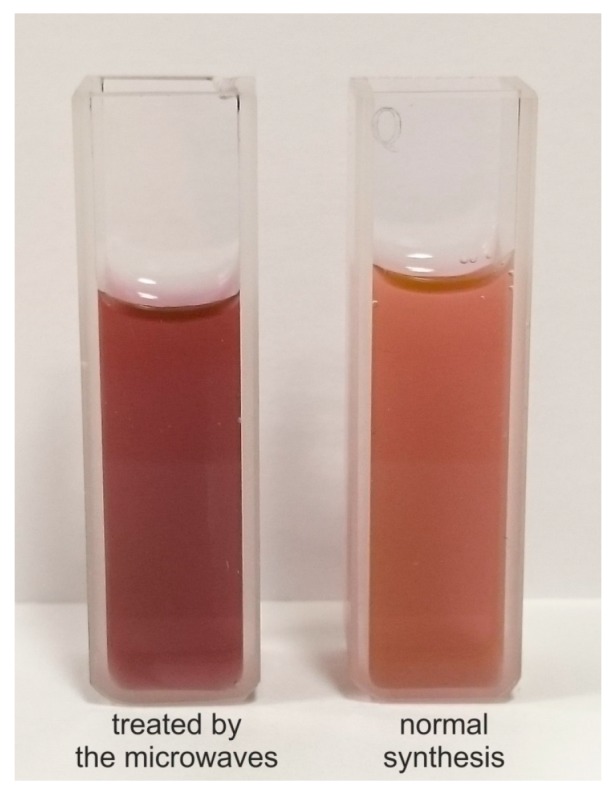
Samples after treatment in the microfluidic reactor and not treated.

**Figure 12:**
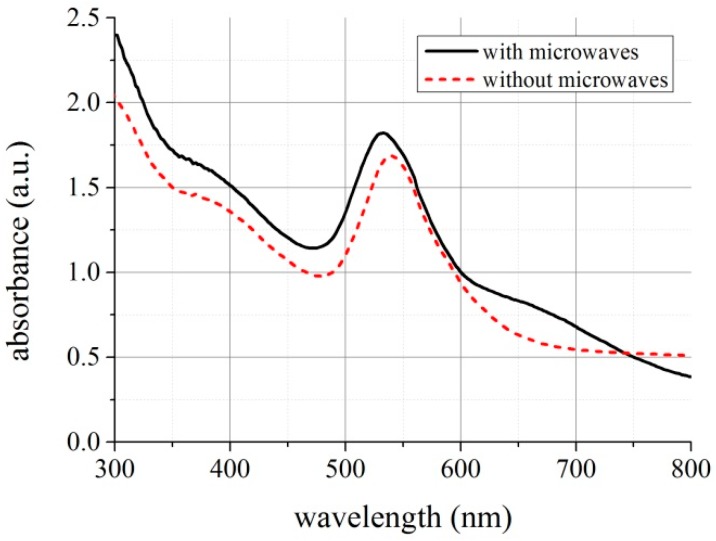
Absorbance of samples with and without microwave treatment.

After each treatment, the sample from the reactor was compared with the reference one. However, absorbance measurements do not show any significant changes immediately after microwave irradiation. We repeated this procedure at different stages of reaction and results were still the same. However, we observed, after a few hours, that samples treated by microwaves differ from samples obtained by means of typical synthesis (see Figure 11). We compared their absorbance and the results are presented in Figure 12. 

**Figure 13:**
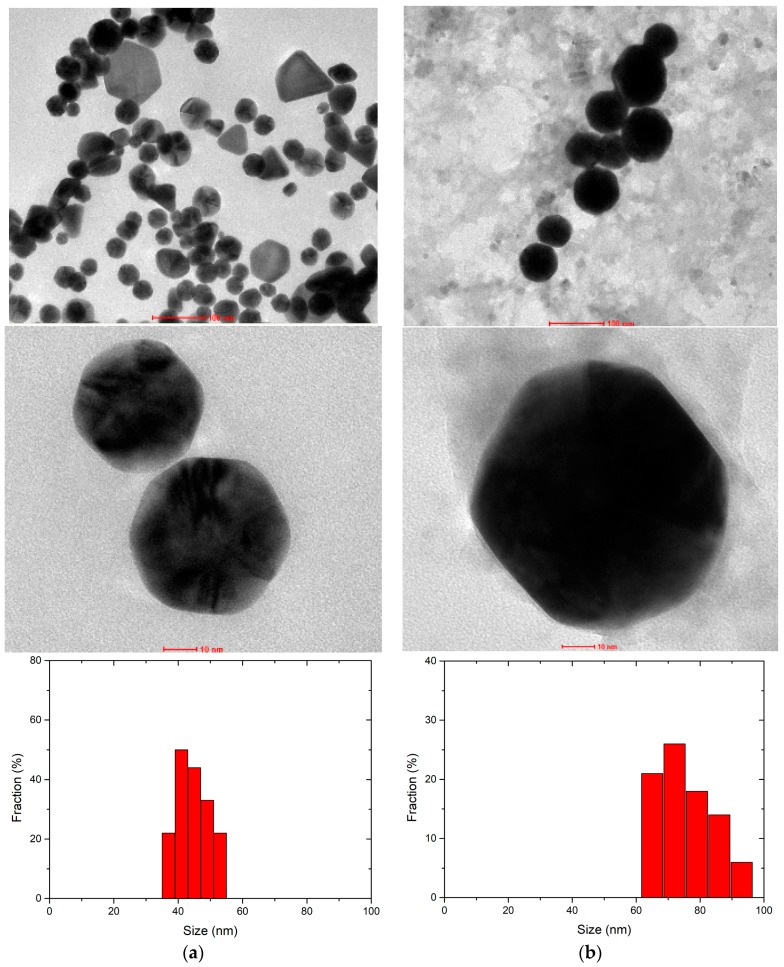
HRTEM images and size distributions of gold nanoparticles obtained: (**a**) with and; (**b**) without microwave treatment.

One may observe that absorbance peak in samples irradiated by microwaves shifts to shorter wavelengths about 8 nm and was 8% higher in magnitude. It indicates that microwaves affected the reaction. In the presence of the microwaves, nanoparticles of smaller dimensions are formed, which, according to the Mie theory [40], causes the shift of the peak towards the shorter wavelength. Gold colloids were tested by high-resolution transmission electron microscope (Figure 13); the images show that in both cases, hexagon-shaped gold nanoparticles with slightly rounded corners were formed. In addition, in the presence of a microwave, field nanoparticles of other shapes are formed and their average size is about 44 ± 9 nm (Figure 13a). During standard synthesis, without microwave field, particles with 76 ± 11 nm diameter are formed (Figure 13b).

The reactions were carried out in an aqueous solution containing only a stabilizing substance, which was polyethyleneimine or another stabilizer of shape and size of nanoparticles is necessary during synthesis because it prevents their agglomeration. In synthesis, no reducing agents were used, which is important because usually gold nanoparticles in aqueous solutions are produced by the reduction of gold ions by reducers such as sodium borohydride, which is highly toxic. According to literature and own work results, synthesis in aqueous solution of polyethyleneimine does not require the use of a reducing agent [41]. In addition, it is possible to reduce gold ions in aqueous solution without reducing and stabilizing agents only subjected to the microwave field [33]. However, in a solution of such composition, particles of about 400 nm are formed, which easily agglomerate.

The reduction of gold ions occurs much faster during the synthesis conducted in the presence of the microwave field. After 5 min, a slight colouring appears, which indicates the formation of gold nanoparticles. The red colour of the solution is a consequence of the plasmonic effect, which is the result of an interaction of electromagnetic radiation on gold nanoparticles [42]. In the case of standard synthesis without the involvement of microwave fields, the progress of the reaction comes after 1.5 h. Conducting the gold ion reduction in the developed microwave reactor improved the rate of formation of gold nanoparticles about 20 times. The particles formed in the microfluidic microwave reactor have more irregular shapes than those produced under standard conditions (without microwave field). In solution exposed to the microwave field, thermal and non-thermal effects occur. As a result of the absorption of microwave energy, the thermal energy of the system increases. In addition, under the influence of frequency-based microwaves, water dipole rotation and ion migration occur. Vo Ke Thanh Ngo and others believe that the formation of nanoparticles with different shapes is due to the intense migration of ions in the microwave field [43].

## 4. Discussion

One may observe that these results obtained in electromagnetic simulations, presented in Figure 4, strongly differ from the measurements. Moreover, the amount of dissipated power can be determined only at selected frequencies. In order to find the explanation for this discrepancy, we used digital radiography for imagining the interior of the reactors (see Figure 5). As can be seen, the main difference between the designed and simulated reactor and fabricated one was the vertical metallization. The vertical metallizations (vias) should be represented in the X-ray image as two lines of bright points. Any additional bright areas indicate defects of the structure. They are visible, especially in 20 mm long reactor (see Figure 5a), whereas 10 and 5 mm long reactors seem to have sufficient quality. Single defects are not a problem, but more than few of them can cause distortion of propagated EM wave. The visible deformations could be caused by the mismatch of the tape and paste shrinkage. As a result, mechanical stresses raised and cracks occurred. Another source of damages could be lamination process, even if the pressure was reduced.

The changes in reactor’s structure explain the unexpected behavior of the measurement results (see, Figure 8) for *f* < 2.3 GHz and *f* > 4.3 GHz). Firstly, our model assumes that reactors are almost perfectly matched, so the power is transferred with extremely low reflections. Unfortunately, degradation of metallizations caused the impedance mismatch. Secondly, analytical model required that the transmission lines are uniform and identical in everything but length. 

Unfortunately, this condition was not fulfilled. One may observe that those lateral metallizations deformed differently in different parts of the reactors, and this surely affected transmission line parameters. For this reason, the best results of power extraction were obtained only for the reactor with 5 and 10 mm length.

When it comes to the synthesis of gold nanoparticles in our experiment, a few hypotheses can be drawn. Firstly, microwave energy was effectively delivered to the sample. Secondly, the elevation of temperature was the key factor responsible for the acceleration of the synthesis reaction rate. However, microwaves have also changed the molecular structure or initiated some processes, which have shown their effect sometime later. It would be useful to investigate the geometry of the synthesized nanoparticles, using, for instance, transmission electron microscopy.

## 5. Conclusions

In this paper, we have presented a device for delivering microwave energy to a liquid sample that flows through a microchannel. The reactor was designed and fabricated in the LTCC technology. It consists of the transmission line buried in the ceramic strip and surrounded by the ground plane metallization. The proposed design of the reactor is presented, as far as we know, for the first time. In addition to this, the use of LTCC technology in this design is also a new achievement. 

We have observed microwave power dissipation in a fluid sample under test. However, the assessment of the amount of absorbed energy was possible only for selected devices and frequencies. The study has shown that these limitations came from technology inaccuracies and the method of measurements. However, it was possible, eventually, to modify properties of the synthesized nanoparticles. Preliminary results presented here are very promising. Therefore, in the next step, the technology and analytical model of the reactor will be improved. This allows us to improve the performance of the microwave reactor and to increase its analytical accuracy.
